# Supplementary material for: Sustained-input switches for transcription factors and microRNAs are central building blocks of eukaryotic gene circuits
Source: Genome Biol. 2013 Aug 23;14(8):R85. doi: 10.1186/gb-2013-14-8-r85 (PMC4054853; doi:10.1186/gb-2013-14-8-r85)
Supplement: Additional file 5 — HTML Browsable Motif Output. Zipped folder containing all WaRSwap and FANMOD motif output, viewable in a web browser. [file gb-2013-14-8-r85-S5.ZIP › HTML_browsable_motif_output/WaRSwap_ath_tair10/sigs_WSWRSLDraw_TAIR10-2500.pvals.heatmaps.html/motif_id_12_010011002_tftype_ath_upstream_-3000_0.html]

```
BG_MODEL = WARSwap
MOTIF_ID = 12_010011002
TF_TYPE = ath
UPSTREAM = -3000_0


PVals
FNR = 0.2	FNR = 0.4	FNR = 0.6	FNR = 0.8
deltaG = 60	0.232	0.099	0	0
deltaG = 70	0.221	0.139	0.003	0
deltaG = 80	0.216	0.162	0.001	0

ZScores
FNR = 0.2	FNR = 0.4	FNR = 0.6	FNR = 0.8
deltaG = 60	0.758	1.349	4.116	5.249
deltaG = 70	0.8	1.127	3.499	5.063
deltaG = 80	0.796	1.048	3.296	4.721

StDevs
FNR = 0.2	FNR = 0.4	FNR = 0.6	FNR = 0.8
deltaG = 60	2302.243	2410.649	2149.068	1471.059
deltaG = 70	2078.551	2173.628	1978.092	1379.021
deltaG = 80	1248.108	1282.548	1152.2	893.933
```
